# No filters, no fridges: a method for preservation of water samples for eDNA analysis

**DOI:** 10.1186/s13104-016-2104-5

**Published:** 2016-06-08

**Authors:** Kelly E. Williams, Kathryn P. Huyvaert, Antoinette J. Piaggio

**Affiliations:** Department of Fish, Wildlife, and Conservation Biology, Colorado State University, Fort Collins, CO 80523 USA; Wildlife Services, National Wildlife Research Center, Wildlife Genetics Lab, USDA, 4101 LaPorte Avenue, Fort Collins, CO 80521 USA

**Keywords:** Environmental DNA, Freshwater, Wild pigs, Noncryogenic, Longmire’s lysis buffer

## Abstract

**Background:**

Advancements in the detection of environmental DNA (eDNA) for detecting species of interest will likely allow for expanded use of these techniques in the field. One obstacle that continues to hinder applications in the field is the requirement of a cold chain of storage for water samples containing eDNA. While eDNA has been successfully preserved using Longmire’s lysis buffer applied to filters, it has yet to be tried with freshwater samples collected for eDNA detection of an invasive species. We tested the utility of Longmire’s solution (100 mM Tris, 100 mM EDTA, 10 mM NaCl, 0.5 % SDS, 0.2 % sodium azide) as an additive to freshwater samples for preservation of eDNA.

**Results:**

Environmental DNA was effectively preserved in 15 mL water samples with Longmire’s solution added; eDNA positive detection was comparable to freezing the samples at −80 °C and occurred out to 56 days at the highest concentration (5 mL Longmire’s solution: 15 mL sample water). Medium and low concentrations of Longmire’s solution added to 15 mL of sample water generally preserved eDNA out to 56 days but not as well as did freezing or application of the highest concentration of Longmire’s lysis buffer. Treatment and degradation time had a significant effect on average DNA concentration of samples, although not the interaction of treatment and time. Perfect detection occurred out to 56 days with the high Longmire’s treatment group but DNA concentration was significantly lower at this time point compared to 28 days.

**Conclusion:**

We conclude that Longmire’s lysis buffer is a viable alternative to cold chain storage that can simplify the collection of eDNA by eliminating the need for filtering and allow more time for sample collection when added at our highest concentration (1 part Longmire’s:3 parts water sample), which could translate to an increase in the chances of detecting a rare or elusive species.

**Electronic supplementary material:**

The online version of this article (doi:10.1186/s13104-016-2104-5) contains supplementary material, which is available to authorized users.

## Background

Analysis of environmental DNA (eDNA), or DNA of a target species captured noninvasively from samples such as soil or water, is a novel method of detecting species of interest in the environment [1–4]. Collection of DNA from water has been successfully used to detect a variety of species from marine and freshwater systems [4–7]. Capture of eDNA from water begins by filtering water samples at the collection site [5, 6] or collecting a water sample and concentrating the eDNA it contains using laboratory methods (chemical and physical) prior to extraction [4, 8]. The preservation of DNA in water samples requires cold storage [9–11] or the addition of a preservative for transportation of filters from the field to the lab. Requiring field personnel to filter and/or manage a continuous cold chain can be expensive, challenging, and time-consuming. Further, freezing and thawing samples prior to analysis reduces DNA viability and thus detection [12]. Longmire’s lysis buffer [13] or ethanol [14, 15] have both been shown to be effective for storage of water filters containing eDNA in the absence of a cold chain.

Longmire’s solution is a lysis buffer that neutralizes cellular components of a sample allowing the DNA to become soluble [16] and to accumulate in the buffer solution over time [17]. Longmire’s solution (100 mM Tris, 100 mM EDTA, 10 mM NaCl, 0.5 % SDS, 0.2 % sodium azide) [16] was originally intended for preservation of tissues for museum collections because such samples are often collected under field conditions without the benefit of refrigeration. Blood samples can be stored in this solution for several years prior to DNA isolation [18]. Longmire’s solution was used to effectively preserve DNA in brain and tail tissue samples from rats for up to 10 months [19] and liver tissue samples from mice for up to 6 months [17]. More recently, Longmire’s solution was used to preserve eDNA captured on water filters [13] for up to 150 days [20] without need for a cold chain. Many forms of lysis buffer have proven effective for noncryogenic preservation of blood and tissue samples in the field [21–23]. Nonetheless, the use of Longmire’s solution as a preservative of eDNA samples from unfiltered freshwater exposed to natural conditions is untested. Here, we present tests of Longmire’s solution for preservation of eDNA from unfiltered freshwater samples. Eliminating the need for time-consuming eDNA capture in the field (filtering) and costly cold chain storage for collecting and transporting water could reduce the time and effort required to collect eDNA samples.

Wild pigs (*Sus scrofa*) are a destructive, invasive species in North America that have widespread negative impacts on ecosystems [24–26]. Management of this species can be challenging when abundance is low, either at the tail end of an eradication effort or in the beginning stages of an invasion process. Successful management of wild pigs requires detection and elimination of individuals before they increase in numbers and spread into new areas [25, 27, 28]. Wild pigs spend time drinking or wallowing in water [25, 29] to thermoregulate and to provide relief from insects and parasites [30–32]. We developed an assay that effectively captures eDNA shed by pigs in turbid freshwater [33]. Application of this assay for surveillance of wild pigs requires sampling from turbid waters (i.e., wallows) under often unfavorable field conditions. Collection of these types of samples needs to be intensive to reach sufficiently large sample sizes needed for detection of wild pigs when abundance is low [7, 34, 35]. Any efficiencies realized in the field, such as eliminating the need to filter each sample or cold chain storage, will reduce the burden on sampling efforts and increase the efficiency of detection surveys. Our goal was to test the effectiveness of Longmire’s solution for preserving unfiltered water samples containing eDNA. Further, we wanted to assess the appropriate volume of Longmire’s solution to add to a 15 mL water sample known to contain wild pig eDNA and to determine the optimal concentration for robust preservation. This study aimed to address the need for an efficient, nonintensive, method of preservation for freshwater samples for optimal detection of eDNA shed by wild pigs.

## Methods

Laboratory work was completed at the USDA-APHIS National Wildlife Research Center (NWRC) in Fort Collins, Colorado, USA. DNA extractions were performed in a lab dedicated to non-invasive and eDNA samples. All PCR and post-PCR procedures were completed in separate rooms. Equipment, benchtops, pipettors, and fume hoods were cleaned with a 10 % bleach solution before and after all procedures.

Water was collected from a 25-gallon tub that served as the water source for a single feral swine sow in captivity at the NWRC/Colorado State University Wildlife Research Facility. Water was collected on June 29, 2015 by submerging a single sterilized 2 L Nalgene bottle and filling it to 1 L.

The 1 L water sample was first mixed using a magnetic stir bar on a stir plate and then subsampled into sixty 50 mL centrifuge tubes in volumes of 15 mL. Subsamples were numbered in order of collection and then randomly assigned to one of five treatment groups using a random number generator. Treatment groups included a positive control where twelve samples were stored at −80 °C (this is an effective method for preserving DNA [36]), a high concentration of Longmire’s solution to sample water (1:3; 5 mL Longmire’s:15 mL sample water), a medium concentration (1:6; 2.5 mL Longmire’s:15 mL sample water), a low concentration (1:15, 1 mL Longmire’s:15 mL sample water), and a no treatment control of 15 mL sample water without lysis buffer or cold storage. Comparison of these groups allowed us to test whether varying amounts of Longmire’s solution affected the preservation of eDNA across the duration of the trial period (56 days).

The no treatment control and Longmire’s solution groups were stored outside in a covered, but not enclosed, area that was exposed to the sun from the West. The tubes were placed upright in a shallow Styrofoam rack that did not completely block incident UV. One half (*n* = 6) of each treatment group was extracted after 28 days and the second half was extracted after 56 days during which eDNA degradation was allowed to occur. During the first 28 days, the treatment groups (excluding the positive control group) were exposed to air temperatures ranging from 12.6 to 33.7 °C. During the second 28 days, air temperatures ranged from 7.3 to 34.2 °C as reported at the Fort Collins Weather Station [37].

DNA was concentrated from the samples via centrifugation [38]. The supernatant was decanted and the DNA pellet was extracted using the DNeasy mericon Food Kit using the 200 mg manufacturer’s protocol (Qiagen). Finally, the elution was cleaned with Zymo IRT columns (additional details [33]). We included a negative control in each set of extractions to monitor for contamination.

Primers and probe for quantitative PCR (qPCR) were used from another study that established best practices for wild pig eDNA capture from turbid water [33]. The qPCR recipe and thermocycling program used are also reported [33]. We used a synthetic internal positive control (ggBlocks® Integrated DNA Technologies) of our target sequence in the D-loop region of *Sus scrofa* to create a standard curve and determined our LOD was one copy/µL. Our qPCR runs fell within the acceptable ranges of an efficiency between 90 and 110 %, a slope between −3.1 and −3.6, and an R^2^ > 0.99 for each plate. Each PCR set included a “no template” negative control including only PCR reagents to monitor for contamination. Each extracted water sample was run in triplicate via qPCR. The criteria for recording a PCR result was that all three replicates of negative controls must be negative. A water sample was considered “positive” if all three qPCR replicates were positive (above our LOD).

We used a Fisher’s exact test to compare the number of samples in which wild pig eDNA was detected (classified as “positive”) between those samples with any lysis buffer treatment and no treatment. We also compared the performance of each concentration of lysis buffer across both time points (28 and 56 days) using Fisher’s exact tests. We used a two-way ANOVA to determine if treatment, degradation time, or the treatment by time interaction had statistically significant effects on DNA concentration. We treated all qPCR replicates within each treatment at each time point as independent of each other for this analysis because we assumed that our mixing the water prior to subsampling homogenized them with respect to the eDNA present in the subsample. Any PCR replicate that fell below our LOD was reported as having a concentration of 0 copies/µL for this analysis. Statistical analyses were conducted in R ×64 3.1.2.

## Results

All positive control (frozen) samples had perfect detection across all qPCR replicates for the duration of the experiment (56 days). We depleted two samples from the low concentration group for 56 days during optimization, leaving us with *n* = 4 for that treatment group.

**Fig. 1:**
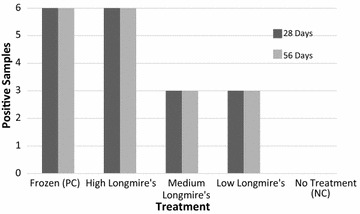
Number of positive samples (3/3 positive qPCRs) per treatment at 28 days (*black*) and 56 days (*grey*). Performance of Longmire’s buffer treatment compared to frozen positive control and no treatment negative control

**Fig. 2:**
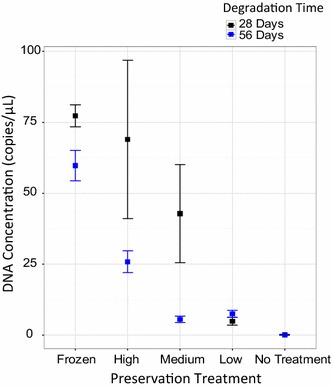
Average concentration of DNA measured per treatment (copies/μL) at 28 days (*black*) and 56 days (*blue*) showing the performance of Longmire’s buffer treatment at preserving DNA. Shown are averages for frozen positive control, high, medium, and low concentrations of Longmire’s, and the no treatment negative control (Calculated from values in Additional file 1). Note that the 28 days mean for “No Treatment” is directly under the 56 days mean as they are nearly equal. *Symbols* represent the mean and *error bars* are the mean ± 1SE

After 28 and 56 days, the qPCR results demonstrated that all volumes of the Longmire’s solution preserved eDNA in our samples significantly better than the “no treatment” group over both time points (Fisher’s Exact test; 28 days: *p* < 0.05, 56 days: *p* < 0.01, Fig. 1). The “no treatment” control group produced only 1 of 3 qPCR positive detections for two samples out of the total six samples taken at 28 days. Because the threshold for a positive detection was complete detection (3 of 3 qPCR results above our LOD), no samples from this treatment group were considered positive. While the raw number of positives using this criterion appeared to differ among lysis treatment groups after 28 days (positive detections: High = 6/6, Medium = 3/6, Low = 3/6) as well as after 56 days (positive detections: High = 6/6, Medium = 3/6, Low = 3/4), these differences were not statistically significant (28 days: Fisher’s Exact test, p = 0.15; 56 days: Fisher’s Exact test, p = 0.13). The highest ratio of Longmire’s solution to water sample had 100 % detection (all positive qPCRs) across both time points (28 and 56 days). However, detection of eDNA in the medium and low ratios of Longmire’s lysis buffer was lower with fewer qPCR positives, suggesting that degradation of DNA had occurred. We found that preservation treatment and degradation time significantly affected average DNA concentrations of the water samples, but the interaction of treatment and time was not statistically significant (Treatment: p < 0.00001, Time: p < 0.05, Treatment × Time: p = 0.14, Fig. 2).

## Discussion

Our results demonstrate that Longmire’s lysis buffer can serve as a viable method for preserving eDNA in unfiltered water samples. However, only the highest concentration (5 mL Longmire’s: 15 mL water) allowed us to detect all samples out to 28 and 56 days (Fig. 1). Although we detected eDNA in all samples in this treatment, DNA concentration declined by 56 days (Fig. 2). We found that the high Longmire’s treatment preserved nearly the same amount of DNA as the positive control (i.e., freezing) after 28 days. Detection of positive samples was lower for the medium and low treatment groups at 28 and 56 days of exposure (Fig. 1). The medium concentration of Longmire’s buffer performed better at 28 days than 56 days but preserved less DNA than the freezing and high Longmire’s solution treatments (Fig. 2). The low Longmire’s treatment preserved a very low amount of DNA compared to the other treatments.

In aquatic systems, environmental DNA is typically dispersed throughout the water body and diluted. We found appreciable variability among the samples in the high and medium Longmire’s treatments at 28 days (error bars, Fig. 2). Inherent heterogeneity of DNA distribution in the water body, regardless of the effort taken to mix the water sample, may be one explanation for this variability. Perhaps the effect of the lysis buffer lysing cells contained in the water samples could explain our quantification of more DNA with the high Longmire’s lysis buffer as compared to the frozen treatment in some samples. Overall, a 1:3 concentration of Longmire’s: freshwater sample was generally as effective as freezing for preservation of DNA out to 28 days.

Our test samples were exposed to extreme summer conditions such as large temperature fluctuations and extended exposure to ultraviolet radiation. These conditions may represent a worst-case scenario for degradation of eDNA in the field. Samples collected for the detection of eDNA from the field will likely be handled more carefully and thus undergo less degradation due to less severe conditions than the samples were exposed to in our study.

As a developing field, advancements in eDNA collection and sample processing are important. Recent reviews and studies have provided optimized methods of eDNA capture from various systems [39, 40]. Longmire’s lysis buffer effectively preserves eDNA on filters [13] without a cold chain and now, based on this study, we know that it is effective in preserving eDNA in unfiltered freshwater samples. Eliminating cold storage of eDNA samples allows for a more efficient method of sample collection that can be used for species detection in monitoring or management activities in the field. For many studies this approach will simplify the collection of eDNA from freshwater systems and allow more time for sample collection, which could mean increasing the chance of detection of a rare or elusive species [13]. This method of preservation may be applicable to other ecosystems but will need to be tested in those systems independently.

## Additional file

10.1186/s13104-016-2104-5 Sample ID and treatment with measured DNA concentration (qPCR) and days of degradation.

## Abbreviation

eDNA: environmental DNA
